# P-776. A Network Meta-analysis of Empirical Antibiotics for complicated Urinary Tract Infections

**DOI:** 10.1093/ofid/ofaf695.987

**Published:** 2026-01-11

**Authors:** Lior Cohen Yatziv, Emily Gilbert, Maria Cristina Vazquez Guillamet

**Affiliations:** University of Illinois Chicago, Chicago, IL; Univesity of Illinois Chicago, Chicago, Illinois; Washington University in St. Louis, St.Louis, Missouri

## Abstract

**Background:**

Urinary tract infections (UTIs) are common bacterial infections causing significant morbidity, mortality, hospital admissions, and health care costs. Rising Antimicrobial Resistance (AMR) in Gram-negative bacilli (GNB), even in community-acquired infections, are making the treatment of UTIs more challenging, underscores the need for developing and evaluating novel therapies and treatment algorithms.

Network Meta Analysis (NMA) is a statistical method that integrates both direct comparisons from head-to-head trials and indirect evidence across studies to simultaneously evaluate and rank multiple treatment options.

We hypothesized that various empirical antimicrobial treatments for complicated UTIs (cUTIs) have similar efficacy and safety. We used NMA to examine this hypothesis.

**Figure:**
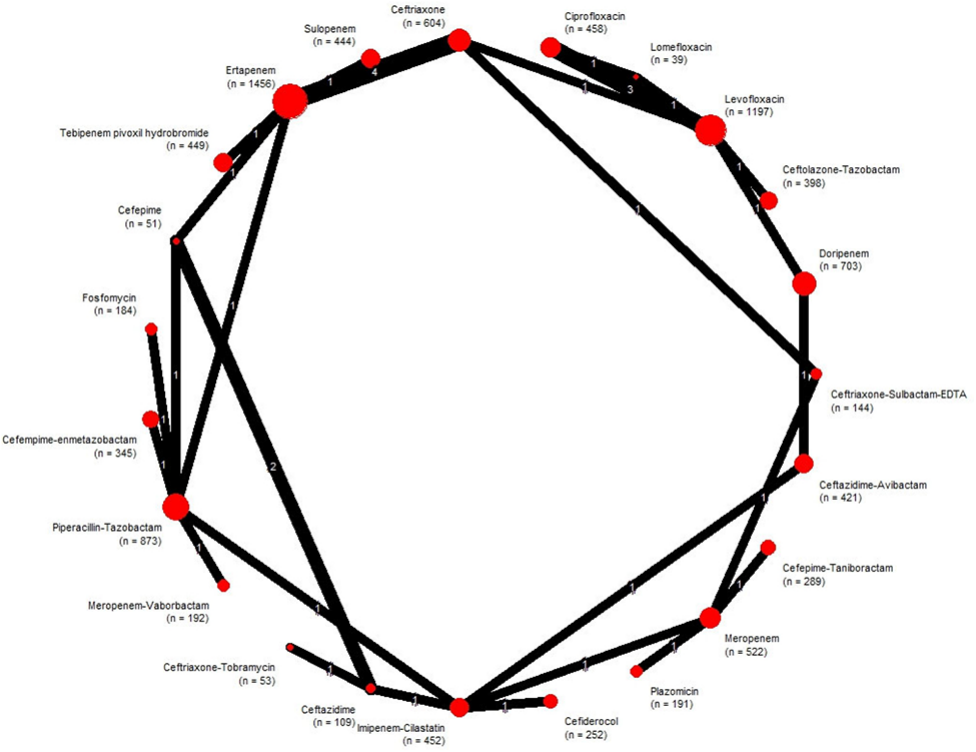
Network geometry for clinical response in randomized trials of empirical antibiotics for complicated urinary tract infection Red circles represent individual antibiotic regimens; circle diameter is proportional to the cumulative sample size for that treatment (n shown in parentheses). Black connecting lines indicate direct head-to-head comparisons; the numeral on each edge denotes the exact trial count. Outcomes refer to clinical response.

**Figure:**
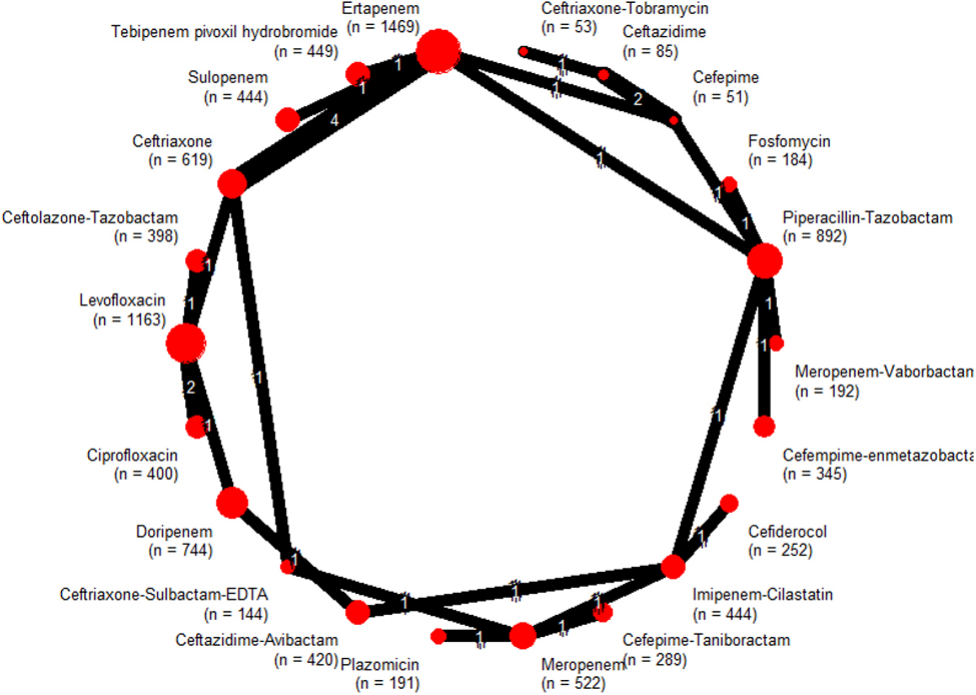
Network geometry for microbiological response in randomized trials of empirical antibiotics for complicated urinary tract infection Red circles represent individual antibiotic regimens; circle diameter is proportional to the cumulative sample size for that treatment (n shown in parentheses). Black connecting lines indicate direct head-to-head comparisons; the numeral on each edge denotes the exact trial count. Outcomes refer to microbiological response.

**Methods:**

PubMed, Embase and the Cochrane Library were searched for randomized controlled trials (RCTs) assessing antibiotic treatments for cUTIs between the years 1990-2025. Searches were developed by a medical librarian using standardized terms and relevant keywords. Primary outcomes included clinical response (CR) – improvement of symptoms, microbiological response (MR) – eradication of bacteria, and drug-related adverse events (AEs). A frequentist random-effects NMA was performed.

**Figure:**
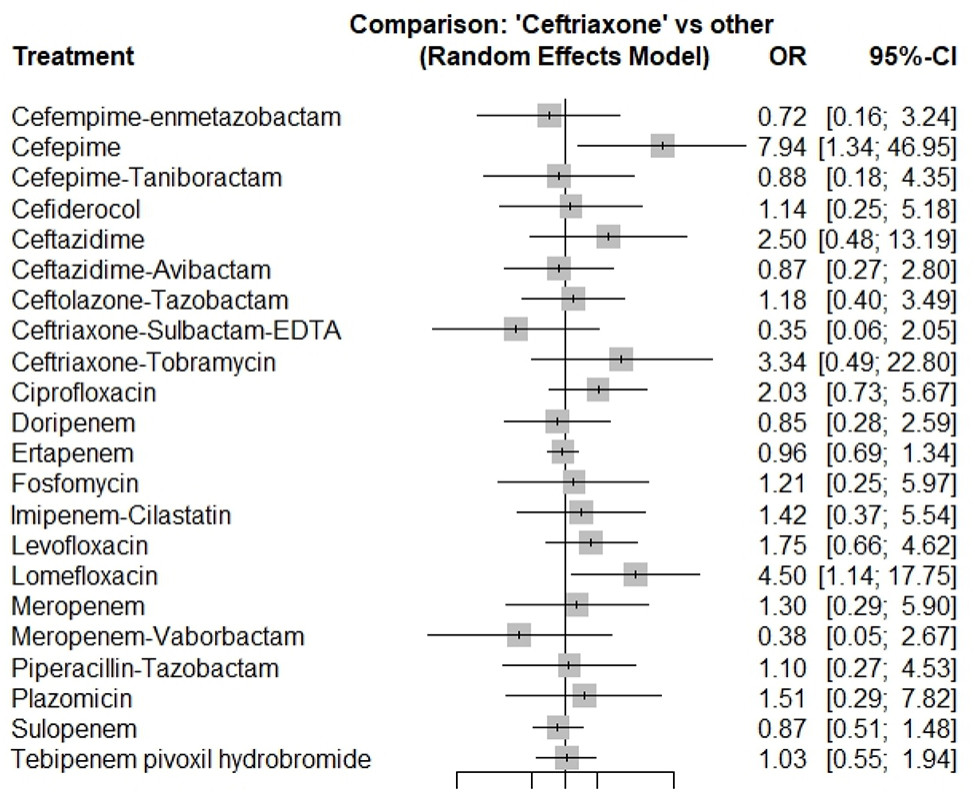
Forest plot of clinical response comparing ceftriaxone with alternative empirical antibiotics for complicated urinary tract infection Log-scaled odds ratios (OR, squares sized by inverse-variance weight) and 95 % CIs for each comparator versus ceftriaxone in complicated urinary tract infection

**Figure:**
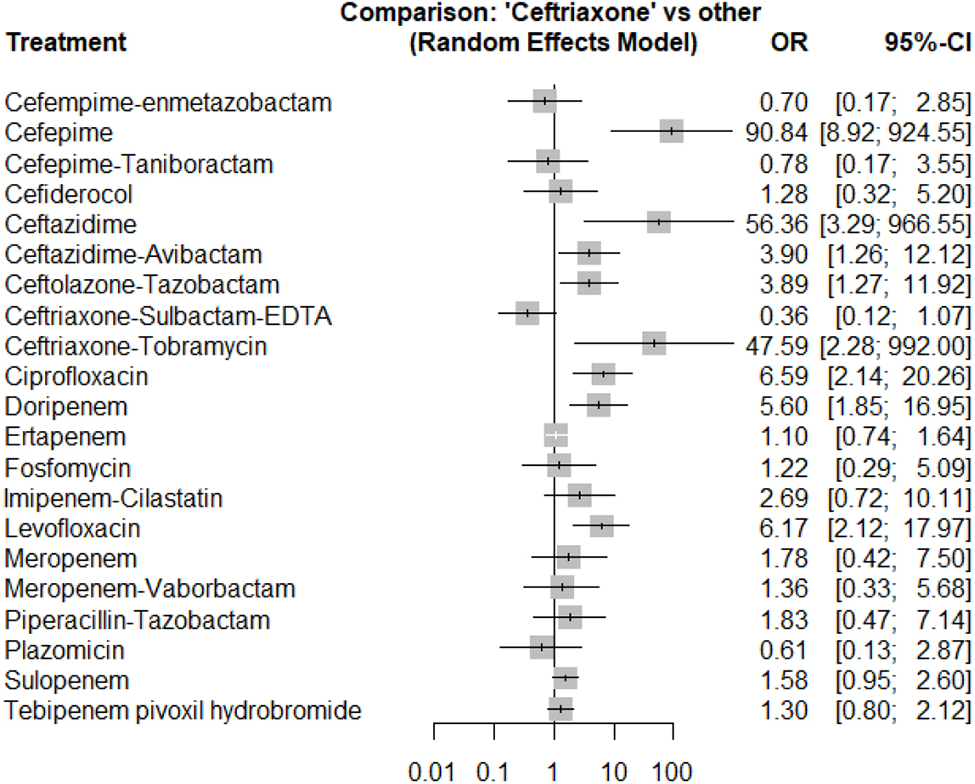
Forest plot of microbiological response comparing ceftriaxone with alternative empirical antibiotics for complicated urinary tract infection Log-scaled odds ratios (OR, squares sized by inverse-variance weight) and 95 % CIs for each comparator versus ceftriaxone in complicated urinary tract infection

**Results:**

We identified 29 RCTs involving 23 antibiotic therapies, including 9,826 patients assessed for CR, 9,750 for MR, and 1,790 for AEs outcomes. Statistical heterogeneity was minimal (Q = 2.57, p = 0.63; I² = 0%). Pairwise comparisons showed no significant difference in CR or MR outcomes compared to ceftriaxone. Safety comparisons against fluoroquinolones revealed no statistically significant differences in serious AEs.

**Conclusion:**

Our NMA demonstrated comparable efficacy and safety across all reviewed antibiotic therapies for cUTIs, with no evidence that novel agents outperform ceftriaxone for empirical treatment. Despite some studies dating back to the early 1990s, including those using cefepime, these antibiotics remain clinically relevant as cornerstone treatments even amid rising AMR rates in GNBs infections. Pending pathogen identification, ceftriaxone provides equivalent CR and MR outcomes, reinforcing its continued role as an effective empirical therapy.

**Disclosures:**

Maria Cristina Vazquez Guillamet, MD, Bionano: Stocks/Bonds (Public Company)|Charisma: Stocks/Bonds (Public Company)|Ocugen: Stocks/Bonds (Public Company)

